# Evidence of acrolein in synovial fluid of dogs with osteoarthritis as a potential inflammatory biomarker

**DOI:** 10.1186/s12891-021-04762-z

**Published:** 2021-10-20

**Authors:** Seth A. Herr, Sarah Malek, Mark C. Rochat, George E. Moore, Jeff C. Ko, Riyi Shi

**Affiliations:** 1 Department of Basic Medical Sciences, College of Veterinary Medicine, Purdue University, West Lafayette, IN USA; 2 Department of Veterinary Clinical Sciences, College of Veterinary Medicine, Purdue University, 625 Harrison St, West Lafayette, IN USA; 3 Department of Veterinary Administration, College of Veterinary Medicine, Purdue University, 625 Harrison St, West Lafayette, IN USA

**Keywords:** Acrolein, Dogs, Osteoarthritis, Synovial fluid, FDP-lysine, acrolein-lysine adduct, MMP2

## Abstract

**Background:**

Acrolein is a known pro-inflammatory toxic aldehyde, propagating cellular damage and tissue inflammation in humans and animal models of various diseases. Osteoarthritis (OA) has a significant inflammatory component; however, presence of acrolein in synovial fluid of joints with OA has not been previously reported. The first aim of this study was to evaluate evidence of acrolein in the synovial fluid of dogs with OA as well as in Control joints. The second aim was to determine if evidence of acrolein can be detected in synovial fluid samples that have been in a frozen state for long periods of time.

**Methods:**

In this pilot clinical study, synovial fluid samples were prospectively collected (i.e., New samples) from a single joint of both clinically healthy (New Control, *n* = 5) and dogs with OA (New OA, *n* = 16) and frozen until the time of analysis. Additionally, frozen synovial fluid samples from a biobank (i.e., Old samples) were used to evaluate ability to detect evidence of acrolein in long-term stored samples (median of 4.89 years) in Old Control (*n* = 5) and Old OA (*n* = 5) samples. Measurements of acrolein in all synovial fluid samples was based on detection of its major protein adduct, *N ε* - (3-formyl-3, 4-dehydropiperidino)lysine (FDP-lysine), using the western blot method. Synovial fluid matrix metalloproteinase 2 (MMP2) was measured in all samples using the western blot method as a positive control of OA inflammation.

**Results:**

Acrolein-lysine adduct was detected in both Control (*n* = 10) and OA (*n* = 21) groups in both Old and New samples. Acrolein-lysine adduct and MMP2 were detectable at a lower level in the Old compared to New synovial fluid samples; however, the differences were not statistically significant (*p >* 0.1). The measured MMP2 levels were significantly higher in the OA compared to Control group samples (*p =* 0.033), but not for acrolein-lysine adduct (*p =* 0.30).

**Conclusions:**

This study confirmed evidence of acrolein in canine synovial fluid of both OA and Control groups. Freezing of synovial fluid for up to 5 years does not appear to significantly affect the ability to detect acrolein-lysine adduct and MMP2 in these samples.

## Background

Endogenous acrolein is one of the cytotoxic aldehydes that is a byproduct of lipid peroxidation or enzymatic oxidation of polyamine metabolites [1]. Acrolein can also be found exogenously as an environmental chemical pollutant from various sources (e.g., incomplete combustion of petrol and plastic, frying oils, and cigarette smoke) [1]. Endogenous acrolein is rapidly deactivated via natural metabolic pathways; however, its increased production under cellular stress conditions can lead to tissue toxicity [2]. Due to inherent instability of the acrolein molecule, more stable protein adducts of this molecule have been used for indirect measurements of in vitro and in vivo acrolein in biological samples [3–5]. Direct and indirect measurements of acrolein have shown elevated levels in various central nervous system, metabolic, hepatic and renal diseases [2, 6, 7]. It has also been shown that exposure to acrolein can exacerbate tissue inflammation and damage in experimental mammalian models of spinal cord injury (SCI) [8]. In dogs, elevation of urinary acrolein in clinical models of SCI has been documented [9]. Anti-acrolein treatments have shown promising results in experimental animal models of SCI by reducing oxidative stress responses in damaged tissues [10–12].

Osteoarthritis (OA) is defined as a disorder involving movable joints characterized by cell stress and extracellular matrix degradation initiated by micro- and macro-injury that activates maladaptive repair responses including pro-inflammatory pathways of innate immunity [13]. The disease manifests first as a molecular derangement (abnormal joint tissue metabolism) followed by anatomic, and/or physiologic derangements (characterized by cartilage degradation, bone remodeling, osteophyte formation, joint inflammation and loss of normal joint function), that can culminate in illness [13]. Osteoarthritis is one of the most prevalent joint disorders in both dogs and humans [14, 15]. In dogs, the most commonly affected sites include knee (i.e., stifle), hip and elbow joints [16]. The most common non-traumatic causes of secondary OA in these joints include degenerative cranial cruciate ligament rupture (CrCLR) in the knee and developmental dysplasia in the hip and elbow joints [17]. The multifactorial etiology and complex pathophysiology of OA has resulted in challenges in timely diagnosis of the disease and lack of currently approved effective treatment options in any species [18, 19]. Investigation of biomolecules involved in the inflammatory process particularly in the synovial fluid of joints with OA, is aimed at discovering novel biomarkers that can be used as diagnostic, predictive and therapeutic biomarkers [20]. Presence of protein-bound and free acrolein in peripheral circulation and urine has been reported in rheumatoid arthritis (RA) in humans but no other form of arthritis [21, 22]. To the authors’ knowledge, evidence of acrolein in synovial fluid of dogs with or without OA has not been previously reported. Dogs are a commonly utilized animal model in OA research [23]; therefore, establishing presence of acrolein in clinical OA can lead to trials further investigating this potential biomarker in in dogs OA. This can have significant translational potential for using dogs for targeted (anti-acrolein) therapeutic trials. The primary aim of this study was to evaluate presence of acrolein in synovial fluid of dogs with OA as well as in healthy control joints. The second aim of this study was to evaluate ability of detecting evidence of acrolein in long-term frozen synovial fluid samples of dogs with OA and healthy controls.

## Methods

### Animals

This study was approved by the Purdue Animal Care and Used Committee (protocol #1804001744111). Informed owner-signed consents were obtained from owners of all dogs enrolled in the study. Due to lack of previously established measurements for acrolein in dogs and the pilot nature of the study the target sample size was to enroll a minimum of 15 dogs in the OA group and 5 dogs in the control group. Prospectively collected synovial fluid samples that were frozen for less than 6 months prior to analysis (referred to as New samples) from this cohort were compared to a bank of samples (*n* = 10; 5 OA and 5 Control) that had been frozen for 4–5 years (referred to as Old samples) to evaluate the effect of long-term freezing on the ability to detect acrolein.

The inclusion criteria for the New samples in the OA group were adult medium to large breed dogs that presented to Purdue Veterinary Teaching Hospital (PUVTH) for OA of one or more joints. Diagnosis of OA was based on initial orthopedic examination by a board-certified surgeon (SM, MR), radiographic evidence of OA based on orthogonal views or computed tomography of the joint, and intraoperative confirmation of OA during joint exploration via arthrotomy or arthroscopy. If more than one joint was affected, the most clinically affected joint that underwent surgical intervention at the time of enrollment was included in the study for synovial fluid sampling. Exclusion criteria for the OA group included OA secondary to trauma or non-OA forms of arthritis in any joint (e.g., septic, immune-mediated), systemic diseases including neurological abnormalities, history of surgical intervention in the affected joint, or use of corticosteroids within a month of enrollment in the study. Inclusion criteria for the New Control group were adult, medium to large breed healthy dogs that were deemed free of any abnormalities based on complete physical, orthopedic and neurological examinations. Exclusion criteria for the New Control group was presence of any systemic illness or history of surgical intervention in the sampled joint.

The Old samples for both OA group and Control groups were randomly selected from a surplus of available stored frozen samples from a completed study at University of Prince Edward Island (UPEI). The Animal Care Committee of the University of Prince Edward Island in accordance with the Guide to the Care and Use of Experimental Animals of the Canadian Council on Animal Care (#11–062) had approved the completed UPEI study and surplus of the synovial fluid samples were available for the current project. The inclusion criteria for Old samples in the OA group and Control group dogs were the same as the fresh samples of OA and Control groups with the exception that OA group dogs were only affected with uni- or bilateral knee OA secondary to CrCLR.

Medical record information for age, breed, gender, body condition score (BCS) based on a 9-point scale [24] were obtained for all groups. Additional recorded information for the dogs in the OA group included documented underlying cause of OA and preoperative radiographic grading of aspirated joints by one observer (SM) using previously established grading schemes for each joint [25, 26].

### Synovial fluid sampling

Dogs in the OA group underwent general anesthesia for surgical intervention relevant to the underlying cause of OA in each joint. The anesthesia protocol was unrelated to the study and based on the discretion of the attending board-certified anesthesiologist for each case. Aseptic arthrocentesis was performed using a 3 ml syringe and 22-gauge needle prior to surgical intervention for each joint. Dogs in the New Control group were recruited from an in-house research colony Purdue University College of Veterinary Medicine. The Control dogs were anesthetized for reasons unrelated to this project and arthrocentesis was performed from both knees using the previously described technique. The knee joint was selected due to ease of access to the joint and ability to obtain higher synovial fluid volume compared to other joints in healthy dogs. The synovial fluid samples obtained from dogs in both OA and Control groups enrolled at Purdue University were centrifuged in EDTA-free tubes at 4 °C for 10 min at 14,000 RPM to remove cells and debris. Thereafter, theses samples were aliquoted in 2 ml cryovials, labeled as New OA and New Control samples, and frozen at -80 °C until analysis. The dog synovial fluid samples used from the UPEI study for the OA group had been collected in the same manner as the PUVTH dogs and Control group samples had been obtained via arthrocentesis of knees immediately after humane euthanasia (for reasons unrelated to the project). The synovial fluid samples from the UPEI study had been maintained in the frozen state at -80 °C after acquisition until analysis, when they were centrifuged, aliquoted and labeled as Old OA and Old Control samples based on their group designation at the time of batch analysis.

### Biomarker analysis

Given the reactivity and short half-life of acrolein, direct in vivo quantification of the molecule is difficult [3–5]. However, acrolein will quickly react with nucleophilic amino acid such as lysine, where acrolein is adducted to lysine and thus can be measured with protein detection methods. The *N ε* -(3-formyl-3,4-dehydropiperidino)lysine (FDP-lysine) is a major protein adduct of acrolein, which is detectable by western blot method [3, 27–29]. In this study, acrolein measurements were based on detecting this acrolein-lysine adduct (i.e., FDP-lysine) using previously established western blot methods [30, 31]. Synovial fluid contains an abundance of protein; therefore, α-1 antitripsin (A1T1) was used as the loading control, since it has previously been shown to have consistent levels between Control and OA synovial fluid samples [32]. Matrix metalloproteinase-2 (MMP2) was used as a positive control marker of inflammation, as it has been shown to be elevated in canine synovial fluid samples with OA [33–36].

Briefly, protein concentrations were measured using the Bicinochoninic Acid protein assay kit (Pierce, Rockford, IL, USA) and SPECTRAmax (Molecular Devices, Sunnyvale, CA). Thirty micrograms of protein with 20% Sodium dodecyl sulfate, β-mercaptoethanol, and 2x Laemmli buffer were loaded to a 15% Tris-HCL gels and electrophoresed at 80 V for 2–3 h providing adequate time for band separation. Proteins were then transferred to a nitrocellulose membrane by electro blotting on ice in 70 V for 1–2 h depending on the protein size in 1x transfer buffer with 20% methanol (Tris-Glycine buffer from BioRad, Hercules, CA, USA). The membrane was incubated in Ponceau stain (Sigma-Aldrich, 6226-79-5). The membranes for MMP2 and A1AT experiments were cut with a razor blade horizontally into strips within the known and desired kDa ranges. This previously used method [31, 37], was used as sample sparing strategy due to limited availability of synovial fluid samples. The membranes for the acrolein detection were not cut due to the previously unknown behavior of this molecule in synovial fluid sample of dogs. Full membranes and cut strips were blocked in 1x casein (Vector, #SP-5020) at room temperature for 10 min, and immunolabeled overnight at 4 °C with each of these primary antibodies: anti-acrolein (StressMarq, SMC-505D), anti-MMP2 (GeneTex, GTX25704), and anti-A1AT (GeneTex, GTX83663). All selected antibodies in this study have been previously verified for reactivity in dogs. After overnight incubation, the membranes were further incubated with either biotinylated anti-mouse or anti-rabbit secondary antibody (Vector, BA-2000, BA-1000) at 24 °C for 45 min, and signal amplified with an ABC-AmP kit for 10 min (Vector, AK-6000). All washes were done with 1x casein. The DuoLux substrate (Vector, SK-6605) immunodetection kit was used for chemiluminescent signal acquisition and the Azure c300 Western blot imaging system (Azure Biosystems, Dublin, CA) was used to image the membrane. A single investigator (SH) ran all samples simultaneously. The AlphaView software (Protein Simple, Version 3.4.0.0. San Jose, CA, USA) was used to quantify the relative signal for each sample’s band for A1T1 (50 kDa), acrolein-lysine adduct (50-75 kDa), and MMP2 (75 kDa) with a reported software-assigned arbitrary value. MMP2 forms multiple bands depending on experimental conditions [38]; in this study only the proenzyme at 75 kDa with the most prominent band was used for analysis. The values for the acrolein-lysine adduct and MMP2 were normalized to A1AT values for each sample using Excel (Microsoft® Excel® 2016, 16.0.5134.000) software.

### Statistical analysis

Descriptive data for normally distributed variables was reported as mean (±standard deviation: SD) and as median (range) for those without a normal distribution. Statistical analysis was performed using STATA SE (v. 15.1) software. Data analyses for all variables (e.g., age, weight, BCS, biomarkers) were accomplished using student’s t-test, Wilcoxon rank sum test, Fisher’s exact test. Evaluation of correlations of MMP2 and acrolein levels with other patient variables (e.g., chronicity of clinical signs, radiographic grades of OA) was performed using Spearman’s correlation test.

## Results

**Fig. 1:**
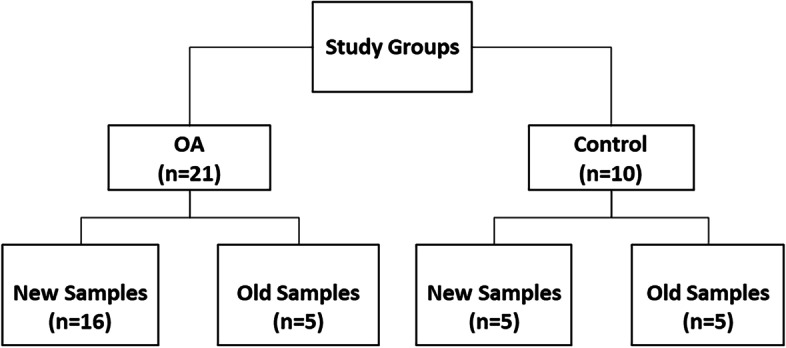
Flowchart of subject recruitment. The number of recruited subjects and samples in each group (OA versus Control) and subgroups (new versus old) based on time elapsed since sample acquisition and freezing until sample analysis. *OA* osteoarthritis

At completion of enrollment, 16 and 5 dogs met the inclusion criteria in the OA and Control groups respectively from which synovial samples were collected and frozen (New samples). Old samples from the bank of frozen synovial fluid samples (*n* = 10) were available from knees of five OA and five Control dogs (Fig. 1). The dogs’ age, weight, body condition score (BCS), and breed distribution details for OA and Control groups are reported in Table 1.

**Table 1:** Characteristics of dogs in osteoarthritis and Control groups

Group	Age (years)	Weight (kg)	BCS (1–9)	Breed (n)	Site and etiology of secondary OA (n)
**OA (*n*** **= 21)**	4.48(±3.53)	35.49(±11.40)	5.79 (4–7)	Labrador Retriever (8) Mixed (5) American Pitt Bull Terrier (*n* = 2) Golden Retriever (2) English Bulldog (1) German Shepherd dog (1) Newfoundland dog (1) Siberian husky (1)	Knee (15) • CrCLR (14) • MPL (1) Elbow (5) • FMCP (3) • FMCP+UAP (1) • OCD (1) Shoulder (1) • OCD (1)
**Control (*n*** **= 10)**	1.43(1.16–1.43)	8.15 (±0.74)	5^a^	Beagle (5) Mixed (4) German Shepherd dog (1)	N/A

Variables with normal distribution include weight and age for the OA group, and weight for the Control group dogs. Data is reported as mean (±SD) or median (range) accordingly. *BCS* Body Condition Score, *OA* osteoarthritis, *CrCLR* cranial cruciate ligament rupture, *MPL* medial patella luxation, *FMCP* fragmented medial coronoid process, *UAP* un-united anconeus process, *OCD* osteochondritis dissecans. ^a^BCS was only available for 5/10 dogs in the Control group and all 5 dogs had a BCS of 5

**Fig. 2:**
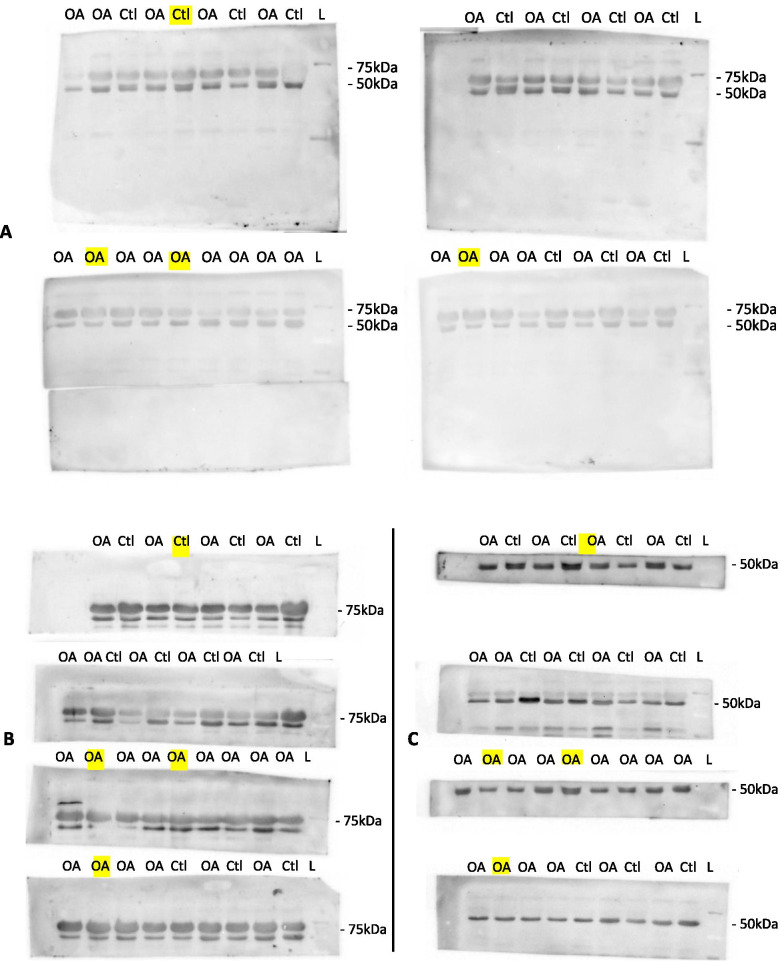
The presented western blots of all samples tested for acrolein-lysine adduct (**A**), MMP2 (**B**) and the loading control, A1AT (**C**). The molecular weight of the bands are shown in kilodaltons (kDa) on the right at each row. The images are of the cut strips of nitrocellulose membrane used as a sample sparing strategy when performing western blots. The highlighted (yellow) samples from four dogs (two OA and one control) were excluded from analysis in the study. These four samples had been obtained from the joints contralateral to those included in the study that were also affected with OA but the study required only one sample per dog to be included in the analysis. *OA* Osteoarthritis group sample, *CtL* Control group sample, *L* molecular ladder, *MMP2* matrix metalloproteinase, *A1AT* α-1 antitripsin

Dogs in the OA group were significantly heavier in weight compared to controls (*p <* .001). The OA group had eight neutered females, 12 neutered males and one intact male, while the Control group included four intact females, five intact males and one neutered male. Overall, the Control group had a significantly higher number of sexually intact dogs compared to the OA group (*P* < .001). The New OA synovial fluid samples collected from PUVTH cohort were from elbow (*n* = 5; 2 right and 3 left), knee (*n* = 14; 5 right and 9 left) and shoulder (*n* = 1, left) joints. In four dogs (i.e., two New OA and one New Control), samples were also obtained from the joint contralateral to that included in the study that were also affected with OA. This was due to concerns that the primary joint that had been sampled may not have yielded adequate samples for the western blot. These samples were run but not included in the analysis since the primary sampled joint had adequate sample volume and the study required only one sample per dog to be included in the analysis. These excluded samples are highlighted in Fig. 2. All Old samples from the biobank were from knee joints (OA group: 3 right and 2 left, Control group: 2 right and 3 left). At the time of batch synovial fluid analysis, the Old samples had been in the frozen state for a mean (±SD) of 4.89 (0.25) years and the New samples had been in the frozen state for a median of 4.50(range, 0.36 to 5.36) months. The underlying cause(s) of OA in sampled joints are summarized in Table 1, with non-traumatic (degenerative) CrCLR (*n* = 14) three of which had concurrent medial meniscal tear in the knee followed by elbow dysplasia (*n* = 5) being most common. The components of elbow dysplasia in these dogs are reported in Table 1.

Acrolein-lysine adduct and MMP2 were detected in both OA and Control group samples. The western blot raw data for acrolein-lysine adduct, MMP2 and A1AT are presented in Fig. 2.

**Fig. 3:**
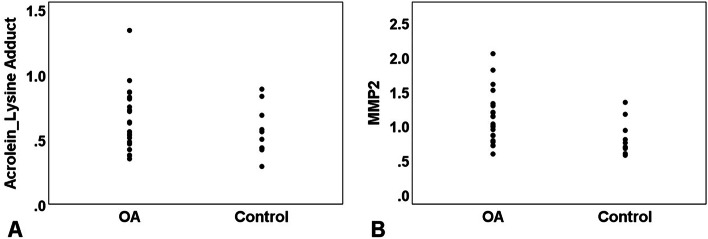
Measured acrolein-lysine adduct (**A**) and measured MMP2 (**B**) for both OA and Control groups. The Y-axis values are arbitrary software assigned values based on strength of western blot bands of acrolein-lysine adduct and MMP2 that have been normalized to the A1AT. *OA* Osteoarthritis, *MMP2* matrix metalloproteinase, *A1AT* α-1 antitripsin

**Fig. 4:**
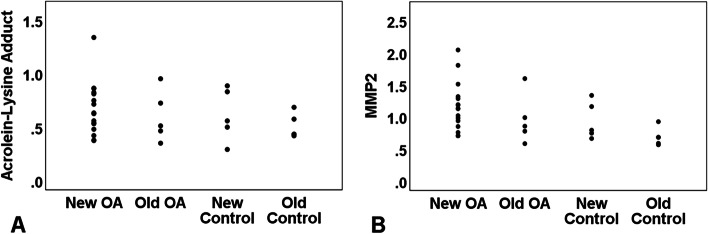
Measured acrolein-lysine adduct (**A**) and MMP2 (**B**) post normalization to negative control (A1AT) are displayed separately as New and Old samples in OA and Control groups. The Y-axis values are arbitrary software assigned values based on strength of western blot bands. *OA* Osteoarthritis, *MMP2* matrix metalloproteinase

Comparison of samples, post normalization to A1AT, showed significantly higher levels of MMP2 (*p* = 0.033) in the OA compared to Control samples but not for acrolein-lysine adduct (*p =* 0.299) (Fig. 3). Mean (±SD) values for acrolein-lysine adduct for the OA group and Control group samples were 0.65 (±0.24) and 0.56 (±0.19), respectively. Mean (±SD) values for MMP2 for the OA group and Control group samples were 1.11 (±0.38) and 0.81 (±0.25), respectively. The measured means for both acrolein-lysine adduct and MMP2 in Old samples were always less than the measurements in New samples in both OA and Control samples, however, these differences were not statistically significant between the Old and New samples in each group(*p >* 0.05) (Fig. 4). Due to presence of higher number of samples from knee joints in both OA and Control groups comparison of biomarker levels were performed in this subgroup as well. The MMP2 levels were significantly different between the knee samples from the OA (*n* = 15) and Control (*n* = 10) groups (*p =* 0.041) but not for acrolein-lysine adduct (*p >* 0.1).

The reported chronicity of clinical symptoms associated with OA in dogs was 3 months (range, 3 days to 18 months). Correlations between the biomarkers (MMP2 and acrolein-lysine adduct) and disease chronicity were evaluated. There was only a weak positive (*r*_*s*_ = 0.335) but not statistically significant correlation (*p =* 0.061) between measured MMP2 and reported chronicity of clinical signs in the OA group.

Radiographic scoring of knees with OA (*n* = 15) based on previously reported grading scheme [26] had scores of 2 (range, 1 to 2) for joint effusion and 2 (range, 0 to 3) for osteophytosis, 0 (range, 0 to 1) for intra-articular mineralization and 2 (range, 0 to 3) for global score. Due to low number of elbow (*n* = 5) and shoulder (*n* = 1) joints, only radiographic grading of knee joints in the OA group were used in evaluating correlations between measured biomarkers and individual grades. Spearman’s rank order correlation showed the radiographic knee OA grade categories (i.e., joint effusion, osteophytosis, intra-articular mineralization and global scores) to be significantly correlated with each other (*p <* 0.05) with the exception of joint effusion and intra-articular mineralization. However, none of these grades had a significant correlation with the measured acrolein-lysine adduct or MMP2 measurements in the OA group.

## Discussion

This is the first study demonstrating evidence of acrolein based on acrolein-lysine adduct (FDP-lysine) detection in synovial fluid of dogs in naturally occurring secondary OA as well as control joints. The indirect measurement of acrolein using acrolein-lysine adduct has been used in other studies [3–5, 21, 39] but had not been utilized in dogs, previously. Ability to detect acrolein-lysine adduct, albeit at lower levels, in synovial fluid samples that had been in a frozen state for up to 5 years, demonstrates its potential value in future studies that aim to utilize samples from biorepositories. The measured acrolein-lysine adduct is the result of strong covalent bonding and is the most stable protein byproduct of acrolein [3]; this may explain its stability in the frozen state. However, further studies are required to evaluate the exact degradation rates of this protein in samples depending on the duration of the frozen state; this is critical if quantification of the protein levels for comparisons is attempted.

Acrolein is capable of initiating and propagating both pain and inflammation by perpetuating oxidative stress and cell death [6, 8, 29]. Other studies have shown other reactive aldehydes, similar to acrolein, to be significantly elevated in synovial fluid and cartilage when using experimentally induced models of OA [40–42]. However, in the current study, despite the observed trend of increased acrolein lysine-adduct in the OA group, the difference between OA and Control groups was not significant. This may be due to the small samples size, variability of clinical samples due to differences in joints and heterogenicity of chronicity and severity of disease in the OA group. Investigation of therapeutic agents facilitating acrolein clearance using aldehyde-scavenging products have already shown significant benefits in alleviating inflammation and improving clinical behaviors in experimental animal models of SCI and multiple sclerosis [43–45]. Anti-acrolein scavengers have not been evaluated in clinical treatment of arthritic conditions such as OA and RA. However, anti-oxidant treatments against similar toxic aldehyde have shown promising results in experimental animal models of OA, and in vitro human RA studies [41, 46]. Anti-acrolein agents already investigated in experimental animal models include dimercaprol, hydralazine, and phenelzine [11, 47–49]. If future studies evaluating acrolein establishes its role in pathophysiology and inflammatory state of OA, these drugs may be considered for future studies evaluating response to targeted therapy in dogs as a translational OA model.

Elevation of synovial fluid MMP2 as a positive control was demonstrated in osteoarthritic joints as a biomarker of inflammation compared to control joints in this study. The use of synovial fluid MMP2 as diagnostic biomarker for canine knee OA has been controversial with some studies showing a positive correlation with OA similar to the findings in this study [35, 50] and some showing none [23]. These variable results may be attributed to heterogenicity of the populations utilized in these studies including the disease severity, etiology of OA, chronicity, analytical methods as well as small sample sizes. Interestingly, synovial fluid MMP2 in human knee OA has shown to be a sensitive biomarker in diagnosing advanced stage of OA from normal joints but not as sensitive in early stages of the disease [51]. This latter trend has also been demonstrated in MMP2 levels in knees with unilateral degenerative CrCLR versus the contralateral knees in dogs that are in early stages of OA [50]. The current study supports the use of MMP2 as a biomarker of inflammation in dogs with OA, however, we were unable to demonstrate a significant correlation between the MMP2 level and reported chronicity of OA. Oxidative stress molecules can directly activate matrix metalloproteinases [40, 52–54]; therefore, anti-acrolein therapies may have the potential to indirectly lead to a beneficial reduction in these inflammatory biomarkers.

Limitations of the current study include the pilot nature with limited sample size, as well as heterogenicity of sampled joints with regards to location (i.e., elbow, knee, shoulder) and etiology of OA. The clinical model of OA in this study was strictly non-traumatic in etiology; therefore, evaluation of presence of acrolein in post-traumatic OA warrants further investigation. In future studies with larger sample size, acrolein levels can be evaluated in specific joints only (e.g., knee) to evaluate the acrolein profile in a range of OA severity while accounting for additional etiologies of OA in that specific joint (e.g., patella luxation, CrCLR, articular trauma) and possible response to medical or surgical interventions. Additionally, levels of acrolein in other forms of arthritis (e.g., immune-mediated arthritis, septic arthritis, RA) warrants evaluation before acrolein can be considered as a candidate diagnostic biomarker of OA. Measurement of acrolein lysine-adduct in this study was based the western blot method that relies on a software-based semi-subjective quantification of the biomarker levels. Use of other quantitative methods such as enzyme-linked immunosorbent assay (ELISA) methodology may provide a basis for developing objective reference ranges for acrolein levels in healthy and disease states.

## Conclusions

This study documented evidence of acrolein in synovial fluid of dogs with OA as well as in control healthy joints by measuring its acrolein-lysine adduct. It is also a proof of concept for using the western blot methodology used herein for detection of acrolein and MMP2 in canine synovial fluid samples, and supports use of samples that have been in a frozen state for an average of 4 years. Future studies are required to investigate the role of acrolein as an inflammatory biomarker in osteoarthritis in dogs.

## Data Availability

The datasets used and/or analyzed during the current study are available from the corresponding author on reasonable request.

## Abbreviations

OA: Osteoarthritis
MMP: Matrix metalloproteinase
SCI: Spinal cord injury
RA: Rheumatoid arthritis
CrCLR: Cranial cruciate ligament rupture
A1T1: α-1 antitripsin
FDP-lysine: *N ε* -(3-formyl-3,4-dehydropiperidino)lysine
PUVTH: Purdue Veterinary Teaching Hospital
UPEI: University of Prince Edward Island
